# Resveratrol enhances A_1_ and hinders A_2A_ adenosine receptors signaling in both HeLa and SH-SY5Y cells: Potential mechanism of its antitumoral action

**DOI:** 10.3389/fendo.2022.1007801

**Published:** 2022-11-03

**Authors:** Sonia Muñoz-López, Alejandro Sánchez-Melgar, Mairena Martín, José Luis Albasanz

**Affiliations:** Department of Inorganic and Organic Chemistry and Biochemistry, Faculty of Chemical and Technological Sciences, School of Medicine of Ciudad Real, Regional Center of Biomedical Research (CRIB), University of Castilla-La Mancha (UCLM), Ciudad Real, Spain

**Keywords:** resveratrol, adenosine receptors, human cancer cells, antitumoral, adenylyl cyclase

## Abstract

Despite great efforts, effective treatment against cancer has not yet been found. However, natural compounds such as the polyphenol resveratrol have emerged as promising preventive agent in cancer therapy. The mode of action of resveratrol is still poorly understood, but it can modulate many signaling pathways related to the initiation and progression of cancer. Adenosinergic signaling may be involved in the antitumoral action of resveratrol since resveratrol binds to the orthosteric binding site of adenosine A_2A_ receptors and acts as a non-selective agonist for adenosine receptors. In the present study, we measured the impact of resveratrol treatment on different adenosinergic pathway components (i.e. adenosine receptors levels, 5’-nucleotidase, adenosine deaminase, and adenylyl cyclase activities, protein kinase A levels, intracellular adenosine and other related metabolites levels) and cell viability and proliferation in HeLa and SH-SY5Y cell lines. Results revealed changes leading to turning off cAMP signaling such as decreased levels of A_2A_ receptors and reduced adenylyl cyclase activation, increased levels of A_1_ receptors and increased adenylyl cyclase inhibition, and lower levels of PKA. All these changes could contribute to the antitumoral action of resveratrol. Interestingly, these effects were almost identical in HeLa and SH-SY5Y cells suggesting that resveratrol enhances A_1_ and hinders A_2A_ adenosine receptors signaling as part of a potential mechanism of antitumoral action.

## Introduction

Cancer is a multifactorial disease characterized by the abrupt uncontrolled transformation of normal cells into cancerous cells and their growth and dissemination throughout the body. Furthermore, cancer is one of the most widely diagnosed diseases and constitutes a leading cause of death worldwide. Despite great efforts, effective treatment against this disease has not yet been found (1). Nevertheless, natural compounds, such as polyphenols, have emerged as promising preventive agents in cancer therapy. Resveratrol (RSV) (3,4,5-trihydroxy-*trans*-stilbene) is a polyphenolic phytoalexin produced by plants under stressful conditions (2). This phytochemical is mainly found in berries, peanuts and grapes. Several *in vitro* and *in vivo* studies have attributed several health benefits to RSV due to its cardioprotective (3), neuroprotective (4) and antitumoral effects, among others. RSV exerts an antitumoral effect in many cancer cell lines, including breast (5), colorectal (6), skin (7), prostate (8) and lung cancers (9). Moreover, various studies have demonstrated that RSV alters many signaling pathways related to the initiation and progression of cancer (10, 11). Resveratrol interacts with many molecular targets which are members of cell survival or cell death signaling pathways, such as caspases (12, 13). Caspases are a family of protease enzymes which play a central role in mediating various apoptotic responses. During the cascade of activation of caspases, caspase-3 activation is involved in the final step. RSV induces caspase-3 activation in human breast cancer cells (14), cervical carcinoma cells (13), and glioblastoma cells (15). Unfortunately, the mode of action of RSV is still poorly understood. Interestingly, one of the signaling pathways that could be related to RSV action is the adenosinergic system (16, 17). Adenosine overproduction occurs in all stages of tumorigenesis, from the initial inflammation/local tissue damage to the precancerous niche and the developed tumor (18). A major source of extracellular adenosine is ecto-5’-nucleotidase (CD73) (19), an enzyme overexpressed in a variety of tumors (20). Adenosine is abundant in the tumor microenvironment (TME), which consists of cancer and immune cells and their surrounding stroma, and it can trigger all four adenosine receptors subtypes (21), which are G-protein coupled receptors (GPCR), named A_1_, A_2A_, A_2B_ and A_3_. Adenosine A_1_ and A_3_ receptors inhibit adenylyl cyclase (AC) activity through G_i/o_, whereas A_2A_ and A_2B_ are coupled to G_s_ protein and can activate AC, thus allowing a reduction or increase in cAMP generation, respectively. These receptors are ubiquitously distributed throughout the mammalian body, including the nervous, cardiovascular, respiratory, gastrointestinal, urogenital, and immune systems as well as in bone, joints, eyes, and skin (22, 23) where they are involved in an extensive list of physiological and pathological processes (24). Moreover, adenosine receptors are expressed at high densities in tumors (25–28). The specific role of adenosine has been described in immunotherapy and points to adenosinergic signaling as a promising target for cancer therapy (25, 29–33). In line with this, adenosine in the TME has been shown to inhibit the anti-tumor function of various immune cells, including cytotoxic T cells and natural killer cells, by binding to cell surface adenosine A_2A_ receptors (34).. The A_2A_ and A_2B_ receptors are primarily responsible for downstream immunosuppressive signaling following the accumulation of intracellular cAMP (35). In turn, A_1_ receptor overexpression promoted cancer cell proliferation *via* the PI3K/AKT pathway while treatment with the specific A_1_ receptor antagonist DPCPX suppresses tumor progression in hepatocellular carcinoma (36). Furthermore, the A_1_ receptor seems to play an antitumor role *via* tumor-associated microglial cells to prevent the development of glioblastomas (37). Finally, A_3_ receptor expression is upregulated in tumor cells and its activation by agonists inhibits tumor proliferation through modulation of Wnt and NF-κB signaling pathways (38). However, in some tumors A_3_ receptor promotes cell proliferation and survival, while in others it triggers cytostatic and apoptotic pathways (37, 39).

Adenosine levels can be fine-tuned by 5’-Nucleotidase (CD73) and adenosine deaminase (ADA) which are responsible for its production and degradation, respectively. Changes in the activity of ADA are detected in patients with various cancer types, suggesting a role in carcinogenesis (40). Presently, multiple CD73 inhibitors are undergoing clinical development for cancer treatment (41).

Our group has previously demonstrated that RSV binds to the orthosteric binding site of A_2A_R and acts as a non-selective agonist for adenosine receptors in rat C6 glioma cells (16). Moreover, we have reported that adenosine receptors may be involved in the antitumoral action of RSV in C6 glioma cells (17). However, these findings have not been further corroborated until now in other tumoral cells.

Therefore, the present study examined the possible involvement of adenosinergic signaling in the antitumoral action of RSV in human HeLa cervical carcinoma cells, as non-neural origin cells, and SH-SY5Y neuroblastoma cells, as a model of a neuronal-like cell. Ee have previously reported some regulatory mechanisms of adenosine receptors in both cell lines (42, 43).

## Materials and methods

### Materials

*Trans*-Resveratrol (RSV, ref. R5010) was purchased from Sigma Aldrich (Madrid, Spain) and calf intestine adenosine deaminase (ADA) (ref. 10102121001) from Roche (Madrid, Spain). A 20 mM RSV stock solution was freshly prepared in 80% ethanol and subsequently diluted in culture medium to the desired concentration. Final ethanol concentration during RSV treatment was 0.8% or less.

### Cell culture

Human HeLa cervical carcinoma and human SH-SY5Y neuroblastoma cells were obtained from the American Type Culture Collection (ATCC). Both cell lines were maintained in DMEM (Dulbecco’s Modified Eagle’s Medium), supplemented with 10% fetal bovine serum (Biowest, ref. S181B-500, Labclinics, Madrid, Spain) and 1% of a mixture of antibiotic-antimycotic (Gibco, USA) and were grown in a humidified atmosphere with 5% CO_2_ at 37°C. HeLa and SH-SY5Y cells were subcultured in a 10 mL Petri dish (Nunc, Roskilde, Denmark). At confluence, cells were detached with trypsin (Tryple Express, Gibco, USA) and were resuspended in complete growth medium and plated in a 6- or 96-well dish (Nunc, Roskilde, Denmark) as necessary.

### Cell viability assay and cell counting

Cell viability was determined using an *in vitro* colorimetric assay kit based on the reduction of tetrazolium salt (XTT) converted to formazan in the presence of an electron-coupling agent, purchased from Roche (Cell Proliferation Kit II, XTT, Roche, Mannheim, Germany). Cells were seeded (5.000 cells per well) in 96-well dishes and exposed to different concentrations of RSV (10, 100, 200, 250 and 500 µM). After 24 h of treatment, cells were incubated with the XTT solution for 60 or 90 min at 37°C. The cleavage of XTT to form an orange formazan dye by viable cells was monitored by reading the absorbance at 475 and 690 nm, according to the manufacturer’s protocol. All samples were run in sextuplicate.

### Wound healing assay

Cells were grown in complete culture DMEM medium to a confluent monolayer. After 24 h, a wound was made by scratching the dish uniformly with a pipette tip, the dish was washed once with complete culture medium, and cells were further grown in complete culture medium in the presence or the absence of RSV. Cell migration was recorded with a digital camera (Leica DFC350FX) attached to a Leica DMI6000B (Leica Microsystems, Wetzlar, Germany) fluorescent microscope using a x 20 HCXPL FLUOTAR objective. Cells were maintained at CO_2_ 5% and 37°C in a stage-top incubation system (PeCon GmbH, Erbach, Germany) for 48 hours during video recording (one image every 2 min). Cell migration parameters were obtained using Wound Healing Automated Cellular Analysis System (ACAS) web-based quantitative image analysis from MetaVi Labs/Ibidi (https://www.metavilabs.com/; Cat# 32000-250, Germany).

### Caspase-3 activity

HeLa and SH-SY5Y cells were detached and counted on a *TC 10™ Automated Cell Counter* (Biorad, Madrid, Spain) after 200 µM RSV treatment. Cells (10^6^ cells) were harvested to measure the caspase-3 activity following the manufacturer’s protocol (Molecular Probes, Barcelona, Spain). Cells were lysed for 30 min at 4°C and centrifuged at 12.000 g for 5 min. The supernatant (50 μL) was collected and added to the P96-black well. 50 μL of a reaction mix containing Z-DEVD, DTT, EDTA, PIPES and CHAPS was then added to each sample. After 30 min of incubation at room temperature protected from light, fluorescence was read at Ex/Em of 340/440 nm, respectively, in a kinetic mode for 4 h. Slope value was used to represent the enzymatic activity. All samples were run in duplicate.

### Nucleus staining

The culture medium was removed, and cells were washed with PBS (pH 7.4). Cells were then fixed with 4% paraformaldehyde for 10 min at room temperature. After three washes for 10 min each in PBS, nuclei were stained with 1 μg/mL DAPI for 10 min, protected from light, and mounted with ProLong Gold antifade reagent (Invitrogen, Madrid, Spain). Nuclei were visualized and quantified by fluorescence microscopy using a DMI6000B microscope and LAS AF software (Leica Microsystems, Wetzlar, Germany).

### Total RNA isolation and preparation of cDNA

Total RNA was extracted from cells using an ABI 6100 Nucleic Acid PrepStation and chemicals according to the manufacturer’s protocol (Applied Biosystems, Madrid, Spain). The A_260_/A_280_ ratio (RNA purity) was in the range 1.8–2.0. RNA concentrations were determined from the A_260_. One microgram of total RNA was reverse transcribed using High-Capacity cDNA Reverse Transcription Kit (Applied Biosystems, ref.4368813) according to the manufacturer’s protocol.

### Quantification of gene expression by real-time PCR

Quantitative real-time RT-PCR analysis was performed with an Applied Biosystems Prism 7500 Fast Sequence Detection System using TaqMan^®^ Universal PCR Master Mix according to the manufacturer’s specifications (Applied Biosystems Inc., Madrid, Spain). The validated TaqMan^®^ probes and primers for A_1_R (assay ID Hs00181231_m1), A_2A_R (assay ID Hs00169123_m1), A_2B_R (assay ID Hs00386497_m1), A_3_R (assay ID Hs00181232_m1), β-actin (assay ID Hs99999903_m1) and 18S (assay ID Hs99999901_s1) were assay-on demand gene expression products from Applied Biosystems. The TaqMan^®^ primer and probe sequences are packaged together in a 20× solution. Human β-actin and GAPDH genes were used as endogenous control. Gene expression assay was carried out following the manufacturer’s indications as described (44). The thermal cycler conditions were as follows: hold for 20 s at 95°C, followed by two-step PCR for 50 cycles of 95°C for 3 s, followed by 60°C for 30 s. Levels of RNA expression were determined using the 7500 Fast System SDS software version 1.3.1 (Applied Biosystems) according to the 2^−ΔΔCt^ method. Briefly, expression results for a gene were normalized to internal control actin or GAPDH relative to a calibrator, consisting of the mean expression level of the corresponding gene in control samples as follows: 2^−ΔΔCt^ = 2^−(Ct receptor gene−Ct actin/GAPDH gene) sample−(Ct receptor gene−Ct actin/GAPDH gene) calibrator^. All cDNA samples were run in duplicate. Results were averaged to produce a single mean quantity value for each mRNA for each sample.

### Preparation of cell homogenate

Control and RSV-treated cells were resuspended in RIPA buffer (50 mM Tris-HCl, 10 mM MgCl_2_, pH 7.4, 1% NP-40, 1% sodium deoxycholate, 0.1% SDS) containing a mixture of protease inhibitors (100 μM PMSF and 100 μg/mL Bacitracin) After homogenization in a Dounce homogenizer (10xA, 10xB) and determination of protein concentrations, samples were stored at −80°C until assays were performed.

### Plasma membrane isolation

Plasma membrane isolation was performed as previously described (16). Control and RSV-treated cells homogenates were centrifuged at 1,000 g and the supernatant was further centrifuged at 12,000 *g* for 30 min. Pellets (plasma membrane) were re-suspended in the homogenization buffer, the protein concentration was determined using the Lowry method, and the samples were then stored. The supernatant was also stored at −80 °C as cytosolic fraction.

### Western blotting assays

To perform Western blotting assays, 30 μg of protein was mixed with loading buffer (0.125 M Tris-HCl pH 6.8, 20% glycerol, 10% β-mercaptoethanol, 4% SDS and 0.002% bromophenol blue), and heated at 60°C for 5 min. SDS polyacrylamide gel electrophoresis (10% SDS-PAGE) was carried out using a mini-protean system (Bio-Rad, Madrid, Spain) with molecular weight standards (Bio-Rad). Proteins were transferred to nitrocellulose membranes, which were washed with PBS containing 10 mM Tris-HCl (pH 7.4), 140 mM NaCl and 0.1% Tween-20, blocked with PBS containing 5% skimmed milk, and then incubated with the primary antibodies at 4 °C overnight. The antibodies used were anti-A_1_R (1:500 dilution, Sigma, ref. A268), anti-A_2A_R (1:500 dilution, Abcam, ref. ab79714), anti-A_2B_R (1:500 dilution, Millipore (Chemicon), ref. AB1589P), anti-A_3_R (1:500 dilution, Sigma, ref. SAB45004), anti-β-actin (1:2000 dilution, Abcam, ref. ab38331) and anti-GAPDH (1:2000 dilution, Abcam, ref. ab8245). After rinsing, the membranes were incubated with the corresponding secondary antibodies (GARPO ref. 170-6515 or GAMPO ref. 170-6516, 1:4000, Bio-Rad, Madrid, Spain). The immunoreaction was visualized using the enhanced chemiluminescence (ECL Prime) detection Kit (GE Healthcare, Madrid, Spain, ref. RPN2236), and the specific bands were quantified in a G:Box densitometer and normalized with β-actin or GAPDH using GeneTools software (Syngene, Bristol, UK).

### 5′-Nucleotidase activity assay

Plasma membrane (30 μg protein) and cytosolic fraction (30 μg protein) from HeLa and SH-SY5Y cells were pre-incubated in reaction medium (50 mM Tris–HCl, 5 mM MgCl_2_ pH 9), at 37°C for 10 min. The reaction was initiated by adding AMP (final concentration 500 μM) and stopped 20 min later by adding 10% trichloroacetic acid. The samples were chilled on ice for 10 min and then centrifuged at 12.000 × *g* for 4 min at 4°C. The supernatants were used to measure inorganic phosphate released as previously reported (45) using KH_2_PO_4_ as Pi standard. The nonenzymatic hydrolysis of AMP was corrected by adding samples after trichloroacetic acid. Incubation times and protein concentration were selected in order to ensure the linearity of the reactions. All samples were run in triplicate. Enzymatic activity is expressed as nmol Pi released/min · mg protein.

### Adenosine deaminase activity assay

Adenosine deaminase (ADA) activity was measured with an enzyme activity assay kit (Abcam ab204695) according to the manufacturer’s protocol (Abcam, Cambridge, UK). The cell homogenate was diluted 1:100 in ADA Buffer Assay in a 96-well plate which was read at Ex/Em = 535/587 nm as a kinetic curve for 30 min. All samples were run in duplicate. Sample values were obtained by interpolation in an inosine standard curve performed in parallel on the same plate as previously described (46).

### Adenosine and related metabolite level quantification by HPLC

Chromatographic analysis was performed with Ultimate 3000 U-HPLC (Thermo Fisher, Madrid, Spain) and data peaks were processed with Chromeleon 7 (Thermo Fisher, Madrid, Spain) as previously described (46). HPLC diode array was used working at a 254 nm wavelength. Purine standards and samples (40 μL) were injected in a C18 column of 4.6 mm × 250 mm with a 5 μm particle size. Two solvents were used for gradient elution: Solvent A—20 mM phosphate buffer solution (pH 5.7), and Solvent B—100% methanol. The gradient was 95% (11 min), 80% (9 min), and 95% (2 min) in Solvent A. The total run time was 22 min with a constant flow rate of 0.8 mL/min at 25°C. Retention times for hypoxanthine, xanthine, inosine, guanosine, and adenosine were 3.5, 3.9, 8.4, 9.4, and 15.5 min, respectively. Purine levels were interpolated from the standard curve constructed with five concentrations of each purine ranging from 0.1 to 500 μM. Data were then normalized to the protein concentration of each cytosolic fraction sample.

### Determination of adenylyl cyclase activity in intact cells

To determine adenylyl cyclase (AC) activity, cells were washed with serum-free DMEM and pre-incubated with 100 μM Ro 20–1724 and 2 U/ml ADA for 15 min at 37 °C to remove endogenous adenosine. AC activity was then induced with various ligands (two wells by ligand) for 15 min at 37 °C in a 24-well plate. The reaction was stopped by adding 0.1 M HCl in absolute ethanol, and cells were then transferred to microtubes. Ethanol was evaporated in a SpeedVac concentrator and the pellet was re-suspended in assay buffer (50 mM Tris-HCl, 4 mM EDTA, pH 7.5) to determine cAMP accumulation, using protein kinase A as cAMP-binding protein and [^3^H]cAMP as radioligand. Standard samples were prepared in the same buffer in a range of 0–16 pmol. The reaction was disrupted by rapid filtration in a Filtermate harvester (PerkinElmer) with filters previously incubated with 3% polyethylenimine for, at least, 30 min. Scintillation liquid mixture was added to filters to count radioactivity. All samples from each well (microtube) were run in duplicate. Two wells from each condition (control, RSV treated) in the 24-well plate were reserved for measuring protein concentrations.

### Statistical and data analysis

Each biological replicate (n value) consisted of cell samples obtained from different passage number at different days. These n values are stated in figure captions. Technical replicates are indicated in the corresponding method section. Statistical analysis was according to Student’s t-test and one-way ANOVA followed by Tukey post-test. Differences between mean values were considered statistically significant at *p* < 0.05. In figures, asterisks represent statistical significance as calculated by Student’s *t*-test (*, *p* < 0.05; **, *p* < 0.01; ***, *p* < 0.001; ****, *p* < 0.0001). The GraphPad Prism 8.4 program was used for statistical and data analysis (GraphPad Software, San Diego, CA, United States).

## Results

### Resveratrol reduces cell viability, migration and cell growth in HeLa and SH-SY5Y cells

**Figure 1:**
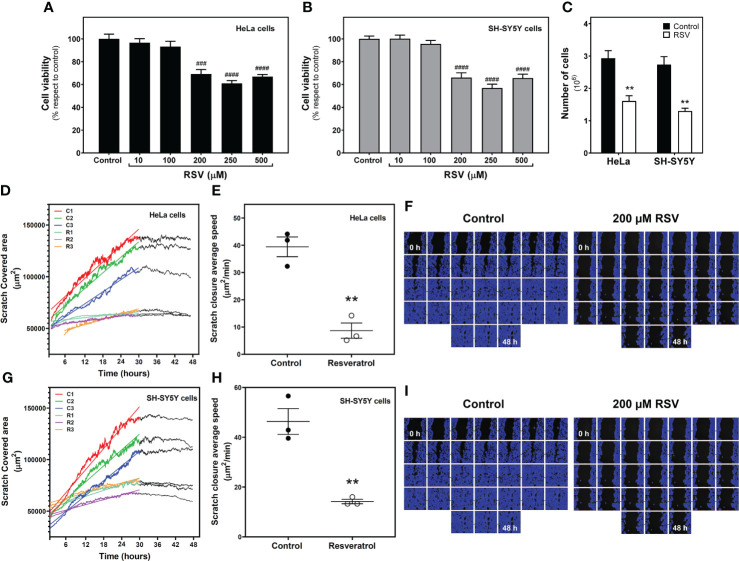
Effect of resveratrol on cell growth. Cell viability was measured with XTT method in HeLa cervical carcinoma **(A)** and SH-SY5Y neuroblastoma cells **(B)** exposed to RSV for 24 hours at indicated concentrations. **(C)** Number of cells counted on a *TC 10™ Automated Cell Counter* after 200 µM RSV treatment for 24 hours. Scratch-covered area **(D)** and scratch closure average speed **(E)** in control and RSV-treated HeLa cells were measured by wound healing assay. **(F)** Representative image series of control and RSV-treated HeLa cells growing for 48 hours after scratch. The blue area represents cell -covered area. A similar analysis was performed in SH-SY5Y cells, with scratch-covered area **(G)** and scratch closure average speed **(H)** measurements. **(I)** Representative image series of control and RSV-treated SH-SY5Y cells growing for 48 hours after scratch. Data are mean ± SEM of four **(A–C)** and three **(D–I)** independent assays; ** *p* < 0.01 significantly different from their corresponding control according to Student’s *t-*test. ### *p* < 0.001 and #### *p* < 0.0001 significantly different from their corresponding controls according to one-way ANOVA followed by Tukey post-test.

To assess the effect of resveratrol (RSV) on human HeLa cervical carcinoma cells and human SH-SY5Y neuroblastoma cells, we first determined the cytotoxicity of this compound by using the XTT assay. Cells were treated for 24h with different concentrations of RSV (10, 100, 200, 250 and 500 µM). Cell viability was significantly decreased by RSV treatment in a concentration-dependent manner in both HeLa (F_(5, 18)_ = 23.19, p < 0.0001, **Figure 1A**) and SH-SY5Y (F_(5, 18)_ = 32.53, p < 0.0001. **Figure 1B**) cell lines, with an estimated IC_50_ value of 130.7 µM and 135.8 µM, respectively. Additionally, 200 μM RSV treatment for 24h significantly reduced the number of cells to half on both cancer cell lines (**Figure 1C**). To further confirm the antitumoral effect of this polyphenol, we evaluated cell migration through a wound healing assay (Supplemental video 1 and 2) in HeLa (**Figures 1D–F**) and SH-SY5Y cells (**Figures 1G–I**). In the absence of Actinomycin D, a cell proliferation inhibitor, RSV treatment at 200 µM strongly and significantly (p < 0.01) reduced scratch closure average speed from 39.41 ± 3.62 to 8.66 ± 2.79 µm^2^/min in HeLa (**Figure 1E**) and from 46.35 ± 5.19 to 14.20 ± 0.90 µm^2^/min SH-SY5Y cells (**Figure 1H**), indicating a cell migration and proliferation reduction of 78% and 69%, respectively. In the presence of 0.05 µg/ml Actinomycin D, scratch closure average speed was 20.34 ± 0.2 µm^2^/min, 51% of that obtained in the absence of this inhibitor (39.41 ± 3.62 µm^2^/min), suggesting that migration and proliferation contribute equally to gap closure. RSV also reduced average scratch closure speed from 20.34 ± 0.2 to 2.98 ± 0.13 µm^2^/min in HeLa cells in the presence of Actinomycin D, which represents a cell migration reduction of 85%. Based on these results, these treatment conditions (200 μM RSV for 24h) were selected for further assays.

### Resveratrol increases caspase-3 activity but does not induce apoptotic bodies

**Figure 2:**
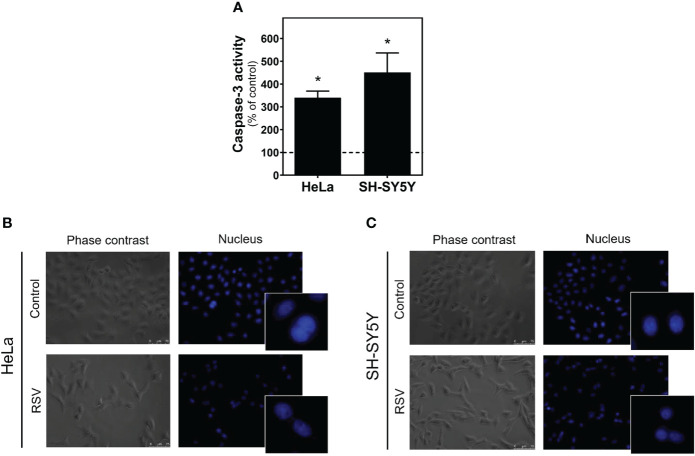
Caspase-3 activity in HeLa and SH-SY5Y cells. **(A)** Increase in caspase-3 activity after 24 hours of 200 µM RSV treatment in both cell lines. DAPI staining of the nucleus in HeLa **(B)** and SH-SY5Y cells **(C)** reveals the absence of apoptotic bodies in control and RSV-treated cells. Data are mean ± SEM of three independent assays; *p < 0.05 significantly different from their corresponding controls according to Student’s *t-*test.

To assess whether reduction in cell viability was due to apoptotic mechanisms, we determined the caspase-3 activity in control and RSV treated cells. As **Figure 2A** shows, significantly increased activity was detected after RSV treatment in both cell lines. However, no apoptotic bodies were found in HeLa (**Figure 2B**) or SH-SY5Y cells (**Figure 2C**).

### Gene expression but no protein levels of adenosine receptors are modulated by treatment with resveratrol

**Figure 3:**
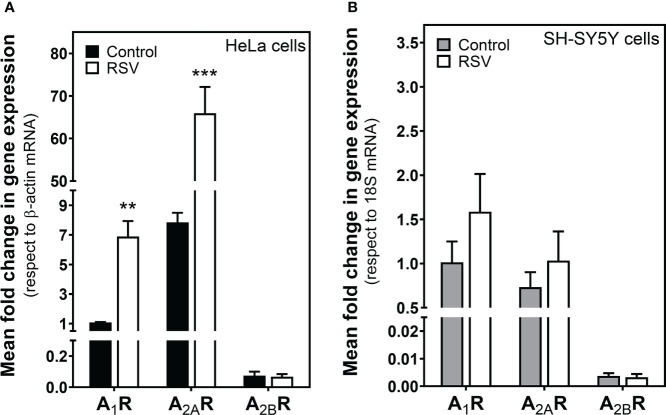
Effect of RSV treatment on adenosine receptor gene expression assayed by quantitative real-time RT-PCR. After total RNA isolation in **(A)** HeLa and **(B)** SH-SY5Y cells, A_1_R, A_2A_R and A_2B_R gene expression levels were detected using TaqMan universal PCR, as indicated in the Materials and Methods section. β-actin and 18S mRNA were used as endogenous control. Data are mean ± SEM of three different samples. **p < 0.01 and ***p < 0.001 significantly different from control cells according to Student’s *t-*test.

We next explored whether the adenosinergic system was modulated by RSV. First, real-time PCR assays were performed in HeLa and SH-SY5Y cells to learn whether RSV treatment modulates adenosine receptors’ gene expression. As **Figure 3A** shows, RSV significantly increased A_1_R and A_2A_R gene expression in HeLa cells, while A_2B_R gene expression was unaltered. However, there were no changes in adenosine receptor gene expression after RSV exposure in SH-SY5Y cells (**Figure 3B**).

**Figure 4:**
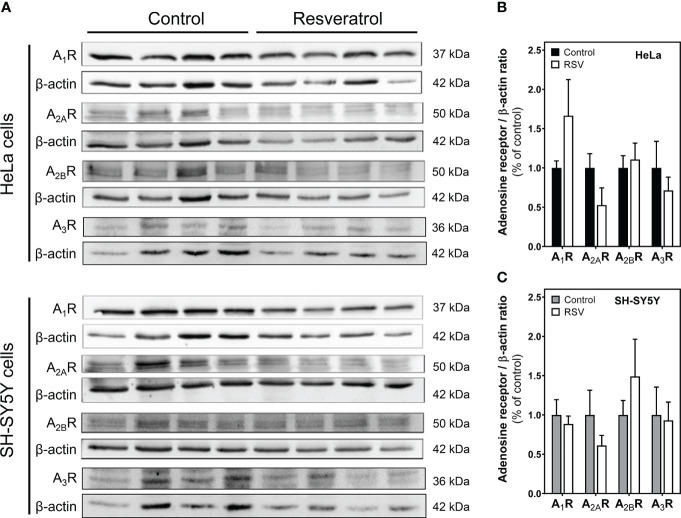
Detection of adenosine receptors. Western blotting **(A)** of adenosine receptors in cell homogenate. Quantification by densitometry of adenosine A_1_, A_2A_ and A_2B_ receptors in HeLa **(B)** and SH-SY5Y cells **(C)**. Data are mean ± SEM of four different control and RSV-treated samples. β-actin was used as a gel loading control.

Next, protein levels of these adenosine receptors were quantified by Western blotting in cell homogenates. Results showed that none of the analyzed adenosine receptors was modulated by RSV in HeLa (**Figures 4A, B**) and SH-SY5Y cells (**Figures 4A, C**). Therefore, no correlation between gene expression and protein levels was detected.

### Resveratrol modulates A_1_R and A_2A_R expressed at the cell surface of intact cells from HeLa and SH-SY5Y cells

**Figure 5:**
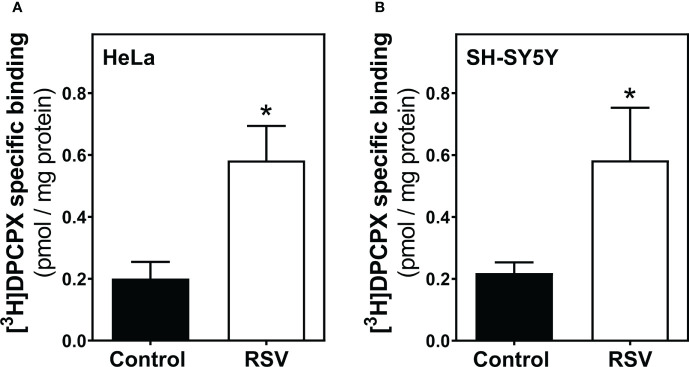
Adenosine A_1_ receptor binding assay. HeLa **(A)** and SH-SY5Y intact cells **(B)** were subjected to a radioligand binding assay to quantify the abundance of A_1_R at the cell surface. A saturation concentration (20 nM) of [^3^H]DPCPX, a selective A_1_R antagonist, was used to determine the specific binding. Data are mean ± SEM of four independent experiments and represented as pmol/mg protein; *p < 0.05 significantly different from their corresponding control according to Student’s *t*-test.

Despite the absence of significant changes in adenosine receptor density quantified by Western blotting, we further examined the modulation of such receptors by radioligand binding assays, which is a particularly sensitive method for the quantification of proteins with a receptor role. First, we observed a significant increase in A_1_R levels after 200 μM RSV treatment in plasma membrane from HeLa (**Figure 5A**) and SH-SY5Y cells (**Figure 5B**), revealing a similar effect of RSV on A_1_R expression in the cell surface of both cell lines.

**Figure 6:**
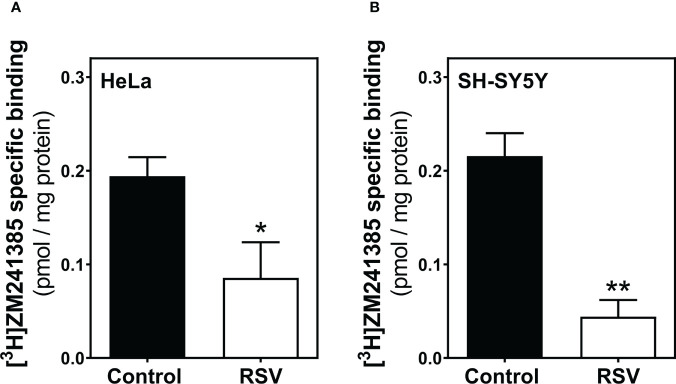
Adenosine A_2A_ receptor binding assay. HeLa **(A)** and SH-SY5Y intact cells **(B)** were subjected to a radioligand binding assay to quantify the abundance of A_2A_R at the cell surface. A saturation concentration (20 nM) of [^3^H]ZM241385, a selective A_2A_R antagonist, was used to determine the specific binding. Data are mean ± SEM of four independent experiments and represented as pmol/mg protein; *p < 0.05 significantly different from their corresponding controls according to Student’s *t*-test.

Next, an appropriate radioligand binding assay was performed to quantify A_2A_R levels at the cell surface of HeLa (**Figure 6A**) and SH-SY5Y cells (**Figure 6B**). Both cancer cell lines showed reduced levels of A_2A_R upon RSV exposure, revealing a similar effect of RSV.

### Resveratrol effect on the adenylyl cyclase activity from HeLa and SH-SY5Y cells

**Figure 7:**
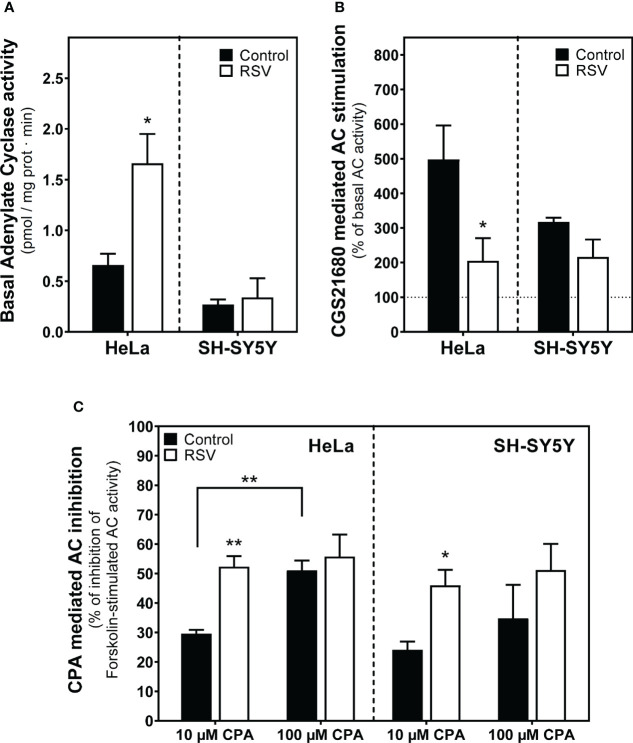
Effect of RSV on adenylyl cyclase activity from HeLa and SH-SY5Y intact cells. **(A)** Basal adenylyl cyclase (AC) activity. **(B)** The A_2A_R-mediated signaling pathway using 10 µM CGS21680, a selective agonist for A_2A_R, represented as the percentage with respect to basal activity of the enzyme. **(C)** The A_1_R-mediated signaling using CPA, a potent agonist for A_1_R, represented as the percentage of inhibition of the 50 μM forskolin-stimulated AC activity. Data are mean ± SEM of three independent samples. *p < 0.05 and **p < 0.01 significantly different from their corresponding controls according to Student’s *t-*test.

Since adenosine A_1_ and A_2A_ receptor levels at the cells surface were modulated by RSV in both cell lines, we next assessed the adenylyl cyclase (AC) effector system. Basal AC activity was significantly increased after RSV treatment in HeLa cells whereas no changes were observed in SH-SY5Y cells (**Figure 7A**). The stimulation of AC activity with 10 µM CGS21680, a selective A_2A_ receptor agonist, decreased in both RSV-treated cell lines but this effect was stronger and significant in HeLa cells. (**Figure 7B**). Finally, the inhibition of AC activity *via* A_1_ receptors was tested by previous stimulation of AC activity with 50 µM forskolin and the absence or the presence of 10 or 100 µM CPA, an A_1_ receptor agonist. In these assay conditions, a greater inhibition of AC activity was clearly observed in RSV treated cells by using 10 µM CPA. At 100 µM CPA the inhibition of AC activity was greater than at 10 µM CPA in control cells and probably strong enough to prevent the detection of even more inhibition in RSV treated cells (**Figure 7C**).

### Resveratrol reduced PKA protein levels in HeLa and SH-SY5Y cells

**Figure 8:**
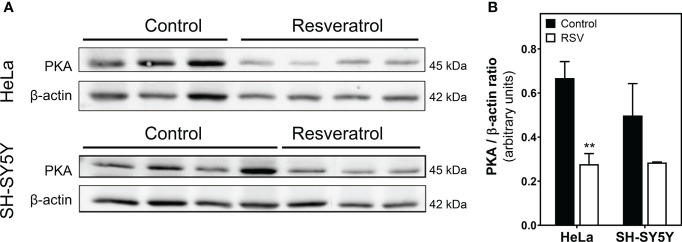
Quantification of PKA protein levels in cell homogenate. **(A)** Control and RSV-treated HeLa and SH-SY5Y cells were analyzed with Western blot. **(B)** Densitometric analysis of the obtained bands. β-actin was used as a gel loading control. Data are mean ± SEM of indicated (three or four) different samples. **p < 0.01 significantly different from their corresponding controls according to Student’s *t*-test.

Next, we analyzed PKA protein levels by Western blotting in cells homogenate. These assays revealed a significantly reduced level of PKA protein in HeLa cells after RSV treatment (**Figure 8A**). However, PKA levels were only slightly decreased but not significantly after RSV treatment in SH-SY5Y cells (**Figure 8B**).

### Modulation of 5′-Nucleotidase and adenosine deaminase activities after resveratrol treatment in HeLa and SH-SY5Y cells

**Figure 9:**
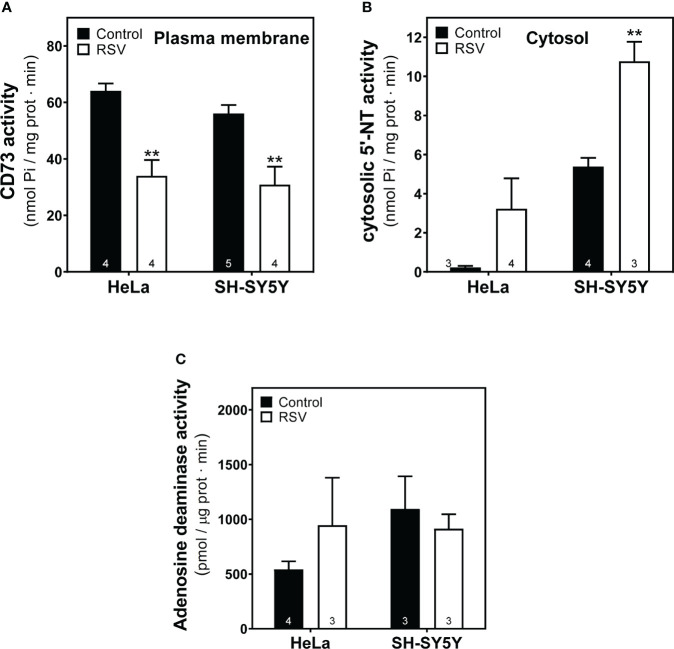
Effect of RSV treatment on adenosine-related enzymatic activities. **(A)** Ecto-5′-Nucleotidase (CD73) activity from the plasma membrane and **(B)** cytosolic-5’-Nucleotidase (NT) activity from cytosolic fraction of HeLa and SH-SY5Y cells were measured as described in the Materials and Methods section. **(C)** Adenosine deaminase activity was measured according to the kit manufacturer’s protocol. Data are mean ± SEM of three to five (indicated within bars) different samples. **p < 0.01 significantly different from their corresponding controls according to Student’s *t-*test.

Next, to further elucidate the effect of RSV on the adenosinergic system, we analyzed the activity of 5’-Nucleotidase (CD73) and adenosine deaminase (ADA), two main enzymes involved in the production and degradation of adenosine, respectively. Data revealed a clear reduction (p<0.01) of the CD73 activity expressed in plasma membrane on both cancer cell lines after RSV exposure (**Figures 9A, B**), whereas increased cytosolic 5’-Nucleotidase activity was found in the cytosolic fraction from both cells, which was statistically significant in SH-SY5Y cells. Concerning ADA activity, RSV treatment did not show any significant effect on this enzymatic activity in either cancer cell line (**Figure 9C**).

### Effect of RSV on adenosine and related metabolite levels

**Figure 10:**
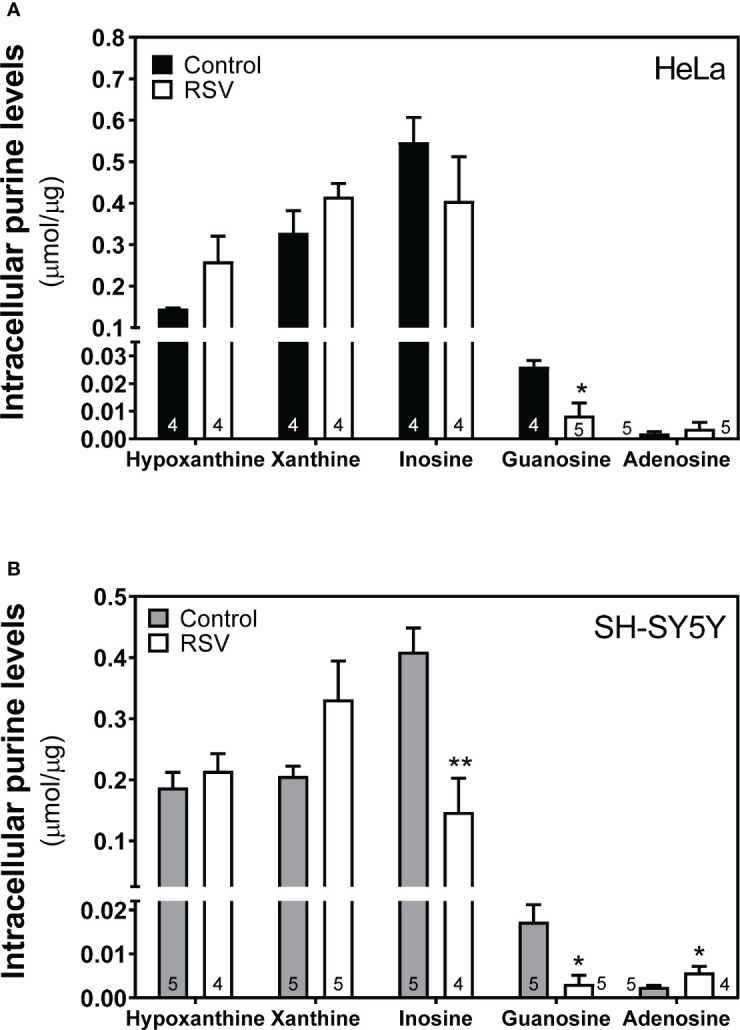
Intracellular adenosine and related metabolites level after RSV treatment. Indicated metabolites were detected and quantified in the cytosolic fraction obtained from HeLa **(A)** and SH-SY5Y cells **(B)** as stated in the Materials and Methods section. Data are mean ± SEM of four or five (indicated within bars) different samples. *p < 0.05, **p < 0.01 significantly different from their corresponding controls according to Student’s *t-*test.

Having observed some significant differences in enzymatic activity related to adenosine metabolism, we next carried out the quantification of intracellular adenosine levels and related metabolites (xanthine, hypoxanthine, guanosine, inosine) using the HPLC method. The profile of these metabolites was very similar in control and RSV-treated cells in both cell lines (**Figures 10A, B**), with significantly lower inosine and guanosine levels and higher adenosine levels in SH-SY5Y cells (**Figure 10B**).

## Discussion

In the present study, we investigated the antitumoral effects of resveratrol in HeLa and SH-SY5Y cells and explored the possible modulation of key components in the adenosinergic signaling pathway. Our results showed that RSV diminished cell viability, migration, and growth, and that different participants on adenosinergic signaling were significantly modulated in both cell lines. Interestingly, the effects of RSV were similar in both cell lines suggesting a common action mechanism by means of which RSV could modulate this signaling pathway (**Scheme 1**).

A large body of evidence has corroborated the antitumoral effect of RSV in both *in vitro* and *in vivo* models of numerous cancer cell types (47–50). RSV can be considered an anti-cancer agent (47) with potential use for cancer therapy (2, 51) and prevention (1) since it affects a variety of cancer stages from initiation and promotion to progression by acting on the diverse signal-transduction pathways that control cell growth and division, inflammation, apoptosis, metastasis, and angiogenesis [reviewed in (2)]. In addition, RSV can reverse multidrug resistance in cancer cells, and, when used in combination with clinically used drugs, it can sensitize cancer cells to standard chemotherapeutic agents (2, 52–54). This effect depends on the dose and tissue (55). Resveratrol inhibits cell viability in a dose−dependent manner in several cervical cancer cell lines, with inhibitory concentration 50 (IC_50_) values in the range of 50–200 µM (C33A 194.6 µM, CaLo 203.9 µM, HeLa 137.1 µM, CaSki 53.5 µM, and SiHa 198.5 µM) (56). We found a similar IC_50_ (ca. 130 µM) in both cell lines analyzed here after 24 h of RSV treatment. This value was ten-fold higher than that achieved with cisplatin after treatment for 24 h (IC_50_ = 16.3 μM) and 48 h (IC_50_ = 14.9 μM) as previously reported by our group in these HeLa cells (57). In human gastric cancer cells these IC_50_ values ranged from 150 to 200 µM [BGC823 200 μM (58), MGC803 127 μM (59)]. Therefore, the growth inhibition activity of RSV appears to be cell-specific, as the IC_50_ differs according to the cell type. In agreement with this, RSV showed higher potency in decreasing the growth of metastatic HeLa and MDA-MB-231 (IC_50_ = 200-250 μM) cells than of low metastatic MCF-7, SiHa and A549 cells (IC_50_ = 400-500 μM) and non-cancer HUVEC and 3T3 (IC_50_≥600 μM) cells after 48 h exposure (60). However, the mode of action of this natural compound remains to be elucidated. The ability to prevent carcinogenesis includes the inhibition of oxidative stress, inflammation, and cancer-cell proliferation, and the activation of tightly regulated cell-death mechanisms [reviewed in (2)]. The molecular mechanisms underlying its chemopreventive and therapeutic activity involve many intracellular targets [reviewed in (61–63)]. Our data indicate that acute exposure to RSV was able to significantly reduce cell viability in a dose-dependent manner and reduce the number of cells. It is still under debate whether RSV induces cell growth inhibition rather than cell death. It has been reported that autophagy was the predominant form of cell death in HeLa and C33A cells, whereas CaSki and SiHa cells die by apoptosis (56). Our results suggest that 24 h of exposure to RSV caused cell growth inhibition since RSV-treated cells showed compromised cell growth and an absence of apoptotic bodies in nuclei. Conversely, the increased activity of caspase-3, an early apoptotic marker, upon RSV exposure suggests that apoptotic pathways might be implicated in the antitumoral effect of RSV in longer exposure to this phytochemical, as previously described in human colorectal cancer cells (64) and human lung adenocarcinoma A549 cells (65). It has been reported that RSV exerts apoptosis by increasing the activity of caspase-9 and caspase-3 while concurrently lowering mitochondrial membrane potential in HeLa cells (66). This is consistent with our previous report in C6 glioma cells, where acute RSV treatment increased caspase-3 activity without apoptotic bodies in nuclei but also elicited cell growth inhibition and cell cycle arrest in the G_1_ phase (17). Furthermore, RSV strongly diminished proliferation and migration ability in HeLa and SH-SY5Y cell lines as reported here, in agreement with studies performed in murine prostate cancer cells (8) and HeLa human cervical cancer cells (13) suggesting that RSV exhibits its antitumoral action in several stages of carcinogenesis. Anticancer agents, such as RSV, may act by modulating cell cycle-associated proteins, such as cyclins, cyclin-dependent kinase (CDK), and CDK inhibitors (67). CDK inhibitors are downstream targets of caspase-3 activation, and loss of these inhibitors can result in the aberrant upregulation of CDKs that have been associated with apoptotic cell death (68). In agreement with this, RSV inhibits proliferation in human colorectal carcinoma cells by inducing cell cycle arrest and apoptosis through thecaspase/cyclin−CDK pathway (69).

We have previously reported how RSV binds to the orthosteric site of adenosine A_2A_ receptors and acts as a non-selective agonist for adenosine receptors (16). Moreover, adenosine receptors, mainly A_1_ and A_3_, seem to be involved, at least partially, in the antitumoral action of RSV in rat glioma C6 cells (17). Results presented herein reveal a clear modulation of different adenosine receptors’ gene expression which are not paralleled by changes in the corresponding protein level, mainly in HeLa cells. The discrepancies between gene expression and density of protein receptors have been previously reported in different tissues by our group (16, 70–72). Similarly, it has been reported that levels of the A_1_ receptor mRNA increase after 6 h of sleep deprivation without a concomitant increase in receptor density (73) and no change in A_2A_ receptor density in cell membranes despite dramatic decreases of receptor mRNA in a murine model of Huntington disease (74). A recent study characterizing human cerebral cortical receptor distribution at the level of mRNA expression as well as protein abundance revealed that most of the comparisons showed no direct relation between protein density and transcriptomic information (75). It has been revealed that a considerable fraction of genes show mRNA-protein expression level discrepancy (76), that is, a rather poor correlation of mRNA and protein levels suggesting that diverse regulatory elements or factors play important roles in explaining the experimentally observed differences between mRNA and protein expression (77, 78). Numerous processes happening on the way from mRNA to protein might account for this discrepancy. Adenosine-to-inosine (A-to-I) editing is the most abundant type of RNA editing in mammals, catalyzed by the protein family called adenosine deaminases acting on RNA (ADAR). Recent genome-wide studies have identified significant global alterations of A-to-I editing levels in various diseases, including cancer (79, 80). Another contributing factor would be microRNAs (miRNAs), which play important regulatory roles by targeting mRNAs for cleavage or translational repression. Recent studies have shown that purinergic surface receptors, including adenosine receptors, as well as ectonucleotidase CD39 and adenosine deaminase ADA2, are subject to miRNA regulation [for a review see (81)]. Interestingly, from a methodological point of view, we have confirmed that radioligand binding assay is a more accurate technique than western blotting to detect and quantify adenosine receptors (in general, GPCRs) at the cell surface. Thus, the clear increased and decreased levels of A_1_ and A_2A_ receptors, respectively, detected in intact living cells by radioligand binding assay can hardly be detected by western blotting in cell homogenates. This also suggests that RSV treatment may affect adenosine receptors trafficking rather than endogenous synthesis or protein degradation, which is in line with agonist-mediated effects on adenosine receptors (82). Additionally, it should be noted that the receptor homo- and heterodimerization widely described in adenosine receptors (83, 84) could also be related to these differences. In line with this, oligomerization of the G protein-coupled receptor CCR5 modify its ligand affinity (85).

Over the past decades, adenosine signaling has emerged as a promising target for cancer therapy. Adenosine, an ATP-derived purine, is abundant in the tumor microenvironment (TME), which consists of cancer and immune cells and their surrounding stroma. The specific role of adenosine has been well described in immunotherapy (25, 29–31). Adenosine can be found at high levels in the TME and can trigger all four adenosine receptors subtypes (21). A pioneering study revealed that A_2A_R protects tumor cells from immune response by T cells and its genetic and pharmacological inhibition reversed that protection (86). Furthermore, CD73 has been widely associated with tumor progression (87–89), prognosis (90–92) and even the outcome of cancer therapy (93) in several types of cancers. In fact, small molecule CD73 inhibitor restores and facilitates the anti-tumor immunity (94). Therefore, the inhibition of A_2A_R/CD73 axis may represent a potential therapeutic strategy against cancer. This is of interest since our study shows that RSV induced a clear decrease in both A_2A_R density and CD73 activity expressed in the cell surface from both human cancer cell lines, which suggests that RSV may be acting through A_2A_R/CD73.

Although both A_2A_R and A_2B_R subtypes and CD73 enzyme have been more intensively investigated in the pathogenesis of cancer (95, 96), we cannot rule out the biological action of A_1_R in tumor cell proliferation. In line with this, activation of A_1_R increased cell viability and reduced apoptotic cells in MCF-7 (97), while in CW2 colon cancer cells this activation led to cell death (98), suggesting a tissue-specific role for A_1_R [eviewed in (99)]. Pharmacological blockade of A_1_R and A_3_R with antagonists partially reduced the antitumoral action of RSV In C6 glioma cells (17). Herein, we have shown that RSV caused overexpression of A_1_R located in the cell surface of both cancer cell lines, which confirms that A_1_R could be related to RSV action.

This modulation of adenosine A_1_ and A_2A_ receptors would have an impact on cAMP/PKA signaling. The cAMP-PKA pathway’s role in the growth of various tumors has been reviewed elsewhere (100). cAMP has become a target for new treatments for cancer and tumoral diseases. However, the exact molecular mechanism by means of which cAMP causes or protects against cancer is still not well understood, since cAMP signaling in cancer cells is affected by the type of cell and its surroundings (101). For instance, human malignancies are linked to the cAMP–PKA–CREB signaling system that, when turned on, can lead to the growth of tumors. Therefore, targeting this pathway may be an effective cancer treatment strategy. Interestingly, the effects of RSV on cAMP-PKA pathway reported here (i.e. higher A_1_R and lower A_2A_R levels leading to both decreased cAMP formation, and lower PKA levels) suggest an attempt to turn off this pathway in order to reduce cell growth.

Extracellular adenosine is produced from AMP by the action of ecto-5’-Nucleotidase (CD73). Then, adenosine is degraded into inosine by ADA or recaptured by the cell (102). As the major enzymatic source of extracellular adenosine, CD73 is a key regulator of cellular homeostasis, stress responses, injury, and disease mechanisms across many tissues (19). It is not surprising that multiple CD73 inhibitors are undergoing clinical development for cancer treatment because adenosine suppresses antitumor immunity (25). Moreover, CD73 is overexpressed in a variety of tumors (20). Since many effects exerted by CD73 on tumors are mediated by its production of adenosine, such effects are dependent on the amount and nature of the adenosine receptors expressed by tumor cells and other cells present in the TME (103). Here, we observed a significant decrease in the plasma membrane CD73 activity after RSV exposure in HeLa and SH-SY5Y cells, which could contribute to a decreased level of extracellular adenosine. However, in HeLa cells, increased cell proliferation and motility were related to CD73 overexpression, but the mechanism was independent of enzyme activity (104).

While extracellular adenosine is a well-established immunosuppressive molecule with a well-described role in carcinogenesis and signaling, intracellular adenosine is involved in many functions such as energy homeostasis and nucleic acid metabolism (105). Among the six cytosolic isoenzymes of 5′-nucleotidases, cytosolic 5’-nucleotidase II (cN-II) preferentially hydrolyzes IMP and GMP, generating inosine or guanosine, over AMP or UMP, whereas cN-I hydrolyzes AMP, generating adenosine, more preferentially than IMP or GMP. However, the contribution of cN-II to intracellular dephosphorylation of AMP has been observed in tissues (e.g., liver or brain) with a very low or undetectable expression of cN-I (106–108). Interestingly, while cN-I is mainly expressed in muscle, cN-II is ubiquitous and highly expressed in cells and organs with high nucleic acid turnover and in tumors (109). We found that RSV differently modulated intracellular adenosine and inosine levels depending on the cell type. Perhaps, the accumulation of intracellular adenosine and the reduced levels of inosine in SH-SY5Y cells could be due to the higher production by cytosolic 5’NT, such as cN-I or cN-II, and lower degradation by ADA, respectively, as can be observed in their corresponding enzymatic activities.

The increase in A_1_R and the decrease in A_2A_R and CD73 observed in this study is consistent with our previous observations in rat C6 glioma cells (17) and mouse brain (70). Moreover, other effects such as reduced cell proliferation, lower cell numbers, increased caspase-3 activity and the lack of apoptotic bodies were also observed in C6 cells. This strongly suggests a common mechanism of action by modulating adenosinergic signaling and contributing to the antitumoral effect of RSV.

To sum up, our data show that RSV overexpressed A_1_R and decreased A_2A_R density and CD73 enzymatic activity found at the cell surface in both human cancer cell lines. This effect was also observed in rat C6 glioma cells (17). T herefore, this study suggests a common molecular mechanism involving the adenosine signaling pathway and sheds light on the biological action of resveratrol and its antiproliferative effect on cancer cells.

## Data availability statement

The original contributions presented in the study are included in the article/**Supplementary Material**. Further inquiries can be directed to the corresponding author.

## Author contributions

Conceptualization, MM; methodology, SM-L and AS-M; formal analysis, SM-L, AS-M and JA; investigation, SM-L and AS-M; writing—original draft preparation, AS-M, MM and JA.; writing—review and editing, MM and JA; funding acquisition, MM. All authors contributed to the article and approved the submitted version.

## Funding

This research was funded by the Ministerio de Ciencia e Innovación (grant PID2019-109206GB-I00), by UCLM (grant 2021-GRIN-31118 cofinanced with the European Union FEDER), and by the Junta de Comunidades de Castilla-La Mancha (JCCM) (grant SBPLY/19/180501/000251) to MM.

## Conflict of interest

The authors declare that the research was conducted in the absence of any commercial or financial relationships that could be construed as a potential conflict of interest.

## Publisher’s note

All claims expressed in this article are solely those of the authors and do not necessarily represent those of their affiliated organizations, or those of the publisher, the editors and the reviewers. Any product that may be evaluated in this article, or claim that may be made by its manufacturer, is not guaranteed or endorsed by the publisher.

## Supplementary material

Scheme 1 Effects of resveratrol treatment in HeLa and SH-SY5Y cells. A potential link between the modulation of adenosinergic signaling and the antitumoral action of resveratrol arises from the indicated effects after 24 hours of treatment with 200 µM resveratrol. These effects were common in both HeLa and SH-SY5Y cells.
