# Supplementary material for: Resveratrol enhances A1 and hinders A2A adenosine receptors signaling in both HeLa and SH-SY5Y cells: Potential mechanism of its antitumoral action
Source: Front Endocrinol (Lausanne). 2022 Nov 3;13:1007801. doi: 10.3389/fendo.2022.1007801 (PMC9669387; doi:10.3389/fendo.2022.1007801)
Supplement: Scheme 1 — Effects of resveratrol treatment in HeLa and SH-SY5Y cells. A potential link between the modulation of adenosinergic signaling and the antitumoral action of resveratrol arises from the indicated effects after 24 hours of treatment with 200 µM resveratrol. These effects were common in both HeLa and SH-SY5Y cells. [file Table_1.docx]

Supplementary Material

**Supplementary Video 1.** HeLa cells growth recording for 48 hours after scratch with a tip during wound healing assay.

**Supplementary Video 2.** SH-SY5Y cells growth recording for 48 hours after scratch with a tip during wound healing assay.
